# Spontaneous splenic rupture complicating primary varicella zoster infection: a case report

**DOI:** 10.1186/s13104-018-3430-6

**Published:** 2018-05-22

**Authors:** Aarthy Uthayakumar, David Harrington

**Affiliations:** 1 University College London Hospitals NHS Trust, 235 Euston Road, London, UK; 2 Charing Cross Hospital, Imperial College London NHS Healthcare Trust, London, UK

**Keywords:** VZV, Splenic rupture

## Abstract

**Background:**

Primary varicella zoster virus (VZV) infection is a common illness, predominantly affecting children. Its course is typically benign, although severe complications have been described. Splenic rupture is an extremely rare and potentially fatal complication of primary VZV infection, with only a handful of cases reported in the literature.

**Case Presentation:**

A 32-year-old Romanian man with no significant past medical history, presented with a 2 day history of sudden onset, worsening generalised abdominal pain and a 1 day history of vomiting. The following day he developed fevers and a generalised widespread erythematous rash consisting of clusters of macules, papules and vesicles at different stages of development. There was no history of sore throat, coryza, arthralgia, myalgia, cough, shortness of breath, weight loss, or night sweats. There was no recent illness and no history of trauma. CT abdomen showed splenic rupture with intra-abdominal haemorrhage. Admission bloods showed anaemia and thrombocytopenia, with haemoglobin 110 g/l and platelets 78 × 10^9^/l. Viral PCR of vesicle fluid from the rash was positive for VZV DNA confirming the clinical diagnosis of primary varicella zoster infection. Viral serology also confirmed recent infection. He was haemodynamically resuscitated, and underwent laparotomy and splenectomy. He was commenced on IV acyclovir and completed a 5 day course. Prior to discharge he was commenced on recommended splenectomy secondary prevention treatment.

**Conclusion:**

There are several reported complications of varicella infection, more commonly in the immunocompromised population. Spontaneous splenic rupture is an unusual complication of primary VZV infection. Here we report the sixth known case in the literature. Splenic rupture should be considered in cases of primary varicella in young adults presenting with abdominal pain.

## Background

Primary varicella zoster virus (VZV) is a ubiquitous virus of the family herpesviridae. Infection is typically seen in childhood, and typically follows a benign, self-limiting course. It is highly infectious, spread by close contact or respiratory droplets, with the secondary infection rate from a household contact of chickenpox as high as 90% [1].

This case highlights a potentially life threatening complication of a common infection caused by an ubiquitous virus, which may easily be missed if not considered. It is the 6th report of this complication developing secondary to VZV, which infects approximately 90% of people by adulthood.

## Case presentation

A 32-year-old Romanian man presented with a 2 day history of sudden onset, worsening generalised abdominal pain and a 1 day history of vomiting. On the day after admission he developed fevers and a generalised rash which was neither itchy nor painful. There was no history of sore throat, coryza, arthralgia, myalgia, cough, shortness of breath, weight loss, or night sweats. There was no recent illness and no history of trauma.

There was no significant past medical history or previous surgery. He had no known history of chickenpox. He took no medications of any kind. He was a heavy smoker, and reported minimal alcohol intake. He was originally from Romania, and had lived in the UK for 7 months. He had no other travel history. He lived alone and had one regular female sexual partner for the past 3 months.

On examination he was alert and comfortable. His pulse was 110 bpm, blood pressure 145/100 mmHg, respiratory rate 24/min, temperature 37.2 °C, oxygen saturations 97% on room air. He had a widespread erythematous rash consisting of clusters of macules, papules and vesicles at different stages of development, worse on upper limbs, torso, and back. The chest was clear to auscultation and the abdomen was generally tender, with a palpable enlarged spleen. There was no guarding or percussion tenderness. Neurological examination was normal.

Admission bloods (see Table 1) showed haemoglobin (Hb) 110 g/l, white cell count (WCC) 3.6 × 10^9^/l, with lymphocyte count of 0.6 × 10^9^/l, platelets 78 × 10^9^/l. C-reactive protein (CRP) was 59 mg/l. Clotting screen, renal function, liver function and amylase were normal. Hepatitis B surface antigen hepatitis C antibody, and HIV (human immunodeficiency virus) 1 and 2 antibody tests were negative. Epstein barr virus (EBV) serology was viral capsid antigen (VCA) IgM negative, VCA IgG negative, and Epstein Barr Nuclear Antigen (EBNA) IgG positive, consistent with infection more than 8 weeks previously. Cytomegalovirus (CMV) IgG was positive, and CMV IgM negative, consistent with previous infection. Serum VZV IgM was negative, serum VZV IgG equivocal (value 0.65 mIU/ml; reference range-negative < 0.65 mIU/ml, equivocal 0.65–0.9 mIU/ml, positive > 0.9 mIU/ml), and polymerase chain reaction (PCR) of vesicle fluid from the rash was positive for VZV DNA and negative for herpes simplex virus (HSV) DNA, confirming the clinical diagnosis of primary varicella zoster infection.

**Table 1:** Patient’s blood test results, virology results including serology (A) and PCR results from vesicle fluid (B), confirming primary VZV infection, and histology summary from splenic tissue (C)

*A) Blood results:*					
Haemoglobin	110 g/l	CRP	59 mg/l	Malaria parasites	None seen
MCV	91.3 fl	Bilirubin	26 μmol/l	EBV VCA IgG	Negative
Platelets	78 × 10^9^/l	Alkaline phosphatase	27 IU/l	EBV VCA IgM	Negative
White cell count	3.6 × 10^9^/l	Alanine aminotransferase	27 IU/l	EBV EBNA IgG	Positive
Neutrophils	2.4 × 10^9^/l	Albumin	35 g/l	CMV IgG	Positive
Lymphocytes	0.6 × 10^9^/l	Sodium	134 mmol/l	CMV IgM	Negative
Eosinophils	0.0 × 10^9^/l	Potassium	4.4 mmol/l	HIV-1 and 2	Non reactive
Blood film	Thrombocytopenia. Stomatocytes seen	Urea	7.8 mmol/l	Hep B surface antigen	Non reactive
INR	1.0	Creatinine	70 μmol/l	Hep B core antibody	Negative
Prothrombin time	11.4 s	Amylase	33 IU/l	Hep C antibody	Negative
APTT	33.0 s	Lactate	1.2	Urine Chlamydia DNA	Negative
APTT ratio	1.10	Bicarbonate	24	VZV IgG	Equivocal
*B) Vesicle fluid investigations*					
VZV DNA	Detected				
HSV DNA	Not detected				
*C) Histology report summary*					
Spleen shows normal histology with subcapsular haematoma and a minor degree of intraparenchymal haemorrhage. The splenic white pulp and red pulp appear normal. No granulomas are seen, there is no evidence of a neoplastic process. In situ hybridisation for EBV is negative and Congo red stains for amyloid deposition is also negative					

**Fig. 1:**
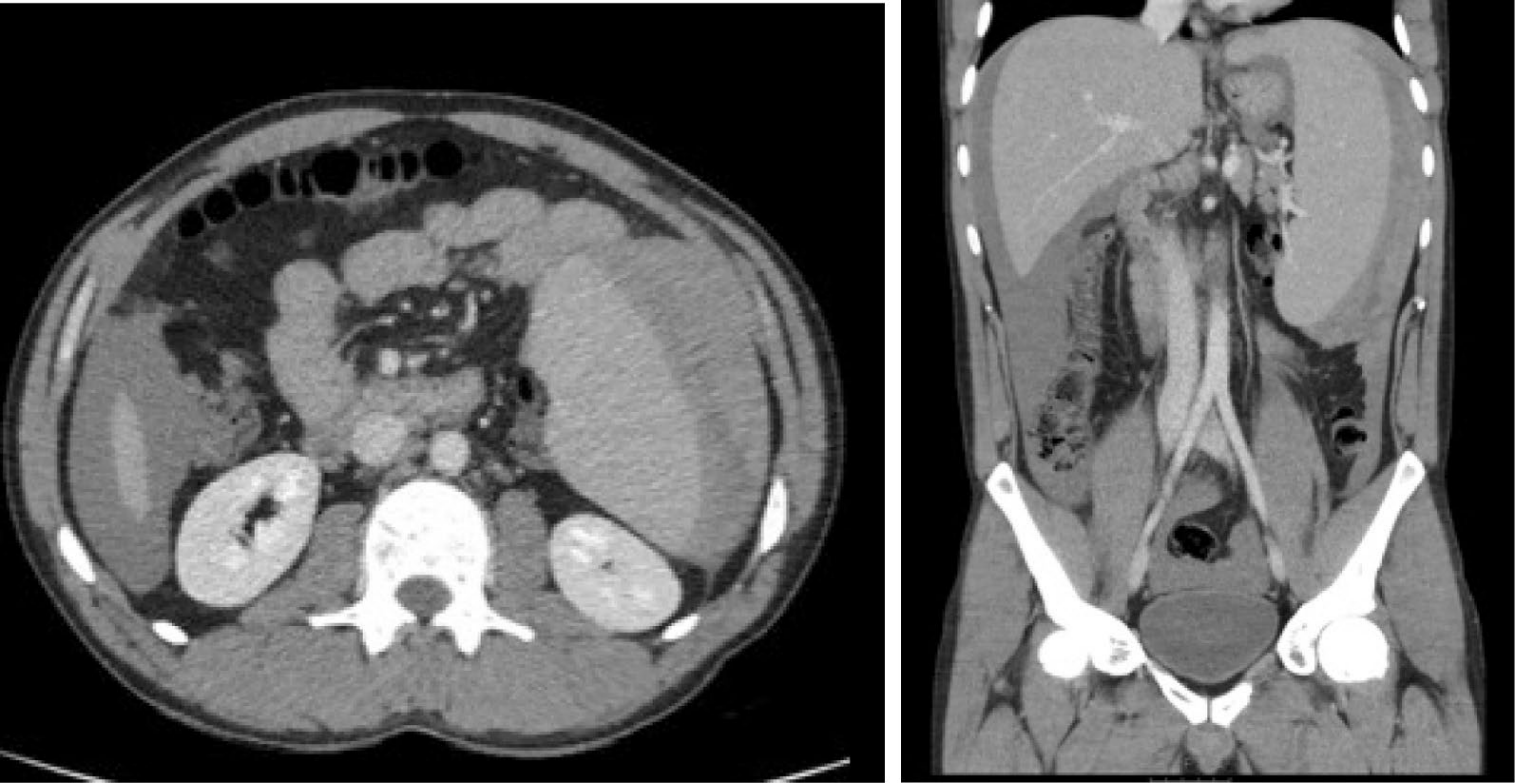
CT abdomen (axial and coronal views) with contrast showing splenic rupture, splenomegaly, and intra-abdominal haemorrhage

Subsequent CT of the abdomen and pelvis showed a splenic rupture of an enlarged spleen (18 cm diameter) with visible capsular haematoma, and extensive high density intra-abdominal free fluid surrounding the spleen and liver, in keeping with haemorrhage (see Fig. 1). The bowel, liver, biliary system, adrenals, pancreas, kidneys and bladder were normal. An ultrasound guided aspiration of the free intra-abdominal fluid revealed frank blood. Subsequent CT angiogram did not identify a bleeding point and illustrated stable appearances of intra-abdominal free fluid.

IV acyclovir 10 mg/kg, 8-hourly and oral co-amoxiclav 625 mg 8-hourly were started on admission. Two units of packed red cells and one pool of platelets were transfused alongside IV 0.9% sodium chloride solution, before undergoing laparotomy and splenectomy on day two of the admission. Surgical findings were of gross peritoneal blood, and ruptured splenic capsule with large adherent haematoma. Histological analysis of the spleen showed normal tissue, with no evidence of amyloid, haematological malignancy or EBV.

He remained alert and haemodynamically stable throughout the perioperative period. He improved clinically and biochemically, with normalisation of the thrombocytopenia and lymphopenia seen on admission. His rash significantly improved, with only a small number of crusted vesicles still present at time of discharge. Co-amoxiclav was stopped after laboratory confirmation of the diagnosis; IV acyclovir was changed to an oral formulation after 4 days, with 5 days acyclovir being given in total. Lifelong prophylactic penicillin V was commenced before discharge, with early follow up booked to administer meningococcus ACWY, conjugate pneumococcus, haemophilus influenzae B, and seasonal influenza vaccines.

He remained well at follow up, with no sign of on-going or recurrent disease.

## Discussion and conclusions

Primary VZV infection causes chickenpox, which is characterised by a prodrome of fever and malaise, which may be absent, followed by a widespread vesicular rash which typically starts on the face and scalp before spreading to the torso and back, and then the limbs. Lesions may be sparse or numerous, and are seen in different stages of development. The infectious period is 2 days before the onset of the rash, and the patient is non-infectious when all lesions have crusted over. Latency is then established in the sensory ganglia, and may reactivate as shingles in later life. Seropositivity is seen in up to 90% of adults [1].

Severe complications are described, and infections can be more serious in the adult or immunocompromised population. Secondary bacterial infections, pneumonitis, meningoencephalitis are well described and are associated with high mortality and morbidity [2]. Cerebellar ataxia is the commonest neurological complication and carries a good prognosis [2]. Transverse myelitis, Guillain-Barré syndrome, thrombocytopenia, haemorrhagic varicella, purpura fulminans, glomerulonephritis, myocarditis, arthritis, orchitis, uveitis, iritis, and hepatitis have also been rarely described [2]. Splenic rupture is an extremely rare complication of primary VZV infection with only a handful of cases reported in the literature. Splenic rupture is more commonly associated with other herpesviridae, notably in glandular fever (infectious mononucleosis) caused by Epstein-Barr virus (EBV), where it complicates 0.1–0.5% of cases [3], and is typically seen in young adults, most frequently men. Splenomegaly and capsular thinning increases the risk of rupture, which may be due to minimal trauma, or occur spontaneously [4]. Other causes of non-traumatic spontaneous splenic rupture include haematological abnormalities such as lymphoma and myelofibrosis, amyloidosis, and other infections including malaria. Spontaneous splenic rupture has a mortality of 12.2% [5], and may require urgent surgical or radiological intervention. In the setting of EBV infection estimates of mortality of spontaneous splenic rupture are between 30 and 100% [6].

Splenomegaly [7], and increased uptake in the spleen seen on Positron emission tomography- computed tomography (PET-CT) [8], has been described in primary VZV infection. Macroscopic splenic nodularity is also described in the setting of primary VZV infection [9]. However, there are only five reports of splenic rupture occurring in the setting of primary VZV infection [9–13]. All cases are reported in young, adult males, as with our case [9–13]. In all cases, abdominal pain preceded the onset of rash, and in all cases but one [9], there was no haemodynamic compromise. The case we present is consistent with these reports, with abdominal pain, likely representing splenic rupture itself, preceding the rash by 48 h. Haemodynamic instability was not present, as with four of the five published reports. Splenomegaly was present as would be expected with a spontaneous splenic rupture, although there is one case in the literature which reported splenic rupture of a normal sized spleen [12]. There was no history of even minimal trauma, as with previous reports.

Spontaneous splenic rupture is an unusual complication of primary VZV infection. Here we report the sixth known case in the literature. Splenic rupture should be considered in cases of primary varicella in young adults presenting with abdominal pain.

## Abbreviations

VZV: varicella zoster virus
HIV: human immunodeficiency virus
EBV: Epstein Barr Virus
VCA: viral capsid antigen
EBNA: Epstein Barr Nuclear Antigen
CMV: cytomegalovirus
PCR: polymerase chain reaction
HSV: herpes simplex virus
PET-CT: positron emission tomography-computed tomography
